# Facial emotion recognition and judgment of affective scenes in Parkinson's disease

**DOI:** 10.1016/j.heliyon.2024.e32947

**Published:** 2024-06-13

**Authors:** Federico Salfi, Stefano Toro, Gennaro Saporito, Patrizia Sucapane, Massimo Marano, Gianluca Montaruli, Angelo Cacchio, Michele Ferrara, Francesca Pistoia

**Affiliations:** a Department of Biotechnological and Applied Clinical Sciences, University of L'Aquila, Via Vetoio, 67100, L'Aquila, Italy; b Research Unit of Neurology, Neurophysiology, Neurobiology and Psychiatry, Department of Medicine, Università Campus Bio-Medico di Roma, Via Alvaro Del Portillo 21, 00128, Rome, Italy; c Parkinson's and Movement Disorder Center, Neurology Unit, San Salvatore Hospital, Via Lorenzo Natali 1, 67100, L'Aquila, Italy; d Department of Life, Health and Environmental Sciences, University of L'Aquila, Via Vetoio, 67100, L'Aquila, Italy

## Abstract

Emotional dysfunctions in Parkinson's disease (PD) remain a controversial issue. While previous investigations showed compromised recognition of expressive faces in PD, no studies evaluated potential deficits in recognizing the emotional valence of affective scenes. This study aimed to investigate both facial emotion recognition performance and the ability to judge affective scenes in PD patients.

Forty PD patients (mean age ± SD: 64.50 ± 8.19 years; 27 men) and forty healthy subjects (64.95 ± 8.25 years; 27 men) were included. Exclusion criteria were previous psychiatric disorders, previous Deep Brain Stimulation, and cognitive impairment. Participants were evaluated through the Ekman 60-Faces test and the International Affective Picture System. The accuracy in recognizing the emotional valence of facial expressions and affective scenes was compared between groups using linear mixed models. Pearson's correlation was performed to test the association between accuracy measures.

The groups did not differ in sex, age, education, and Mini-Mental State Examination scores. Patients showed a lower recognition accuracy of facial expressions (68.54 % ± 15.83 %) than healthy participants (78.67 % ± 12.04 %; p < 0.001). Specifically, the PD group was characterized by lower recognition of faces expressing fear, sadness, and anger than the control group (all p < 0.020). No difference was detected for faces expressing disgust, surprise, and happiness (all p ≥ 0.25). Furthermore, patients showed lower accuracy in recognizing the emotional valence of affective scenes (66.75 % ± 14.59 %) than healthy subjects (74.83 % ± 12.65 %; p = 0.010). Pearson's correlations indicated that higher accuracy in recognizing the emotional facial expressions was associated with higher accuracy in classifying the valence of affective scenes in patients (r = 0.57, p < 0.001) and control participants (r = 0.57, p < 0.001).

Our study suggested maladaptive affective processing in PD, leading patients to misinterpret both facial expressions and the emotional valence of complex evocative scenes.

## Highlights

• Emotional dysfunctions are considered relevant non-motor symptoms in PD.
• We report a lower recognition of emotional facial expressions and evocative scenes.
• Poor performances in emotional recognition may contribute to emotional disorders.

## 1 Introduction

Growing evidence suggests that basal ganglia (BG) influence both motor control and emotional regulation [1,2]. They have been traditionally known to control voluntary behavior, especially goal-directed actions [3]. However, to select appropriate actions and encourage motor habit learning, BG also process emotional stimuli as a drive to adapt motor behaviors to the environment. The ventral subregions of the BG, formed by the nucleus accumbens and the ventral pallidum, are specifically linked to structures of the limbic system involved in perception, motivation, and memory [2]. While contributing to movement selection, BG might also influence emotional control. Cortico-basal ganglia networks support the switch in behavioral control from actions to habits [3]. At the same time, the crossroad between BG and the limbic system may drive the switch from goal-directed behaviors to automatic action tendencies and vice versa. Limbic information might be processed separately from, or jointly with, motor information. A joint treatment would make emotions subserving the initiation and elaboration of behavioral sequences, either by triggering behaviors in response to environmental events or modifying the behavior execution according to the emotional context of actions [2].

Emotional involvement in humans arises from various situations. We receive emotional signals from different sources, e.g., looking at people's faces or observing natural scenes [4]. Facial expressions are essential in perceiving of other's emotional states, while everyday life evokes subjective emotions of various valence and intensity. Emotion recognition can be empirically investigated through different methods, including the Ekman Faces Test and the International Affective Picture System (IAPS), to assess the ability to recognize emotions from faces and real-life pictures. Impaired facial emotion recognition has been detected in experimental settings [5] and pathological conditions [6]. Similarly, a decreased accuracy in recognizing scenes-evoked emotions has been identified in various pathological conditions [7]. Patients with Parkinson's disease (PD), a neurodegenerative disorder affecting BG, have been widely investigated through the Ekman Faces Test with consistent results [8,9]. Conversely, no data about responses to IAPS pictures are available for the same population.

A generalized poorer emotion recognition performance might be expected for different reasons. On the one hand, as voluntary mimicry is involved in the explicit recognition of emotional facial expressions [10], the hypomimia of PD patients, also known as facial masking, might affect the perception of other's emotional states when expressed by faces. On the other hand, PD patients are likely to suffer from emotional dysfunctions of various nature, recently classified among the non-motor symptoms of PD. The BG play a crucial role in the pathophysiology of PD due to their involvement in motor control [11]. The degeneration of dopaminergic neurons in the substantia nigra, which impacts the functioning of the BG, leads to motor symptoms such as bradykinesia, rigidity, and tremor. Moreover, previous studies suggested that the BG, especially the subthalamic nucleus (STN), seem to be directly implied in emotional processing [12]. Electrophysiological studies in PD patients with deep brain stimulation (DBS) have demonstrated that the STN responds to emotional stimuli, with significant activity observed in the alpha frequency band during exposure to both visual and auditory emotional cues. For instance, studies by Kühn et al. [13] and Brücke et al. [14] found that valenced stimuli, whether pleasant or unpleasant, elicited larger alpha band event-related desynchronizations in the STN compared to neutral stimuli. This indicates that the STN is actively involved in processing the emotional valence of scenes. The strong anatomical and functional ties between BG and the limbic system may furtheraccount for the emotional dysregulation in PD. The anatomical overlap between the ventral BG portions and the ventral subcortical structures of the limbic system, the presence of direct afferences to BG from structures associated with the limbic system, and the role of dopamine in BG activities (largely controlled by subcortical limbic structures) may contribute to explain any emotional dysregulation in PD.

Moreover, a growing body of research has proposed amygdala hypofunction and its altered functional connectivity with other brain regions as a core component of dysfunctional responses to emotional face stimuli in PD [15]. Functional alterations in the amygdala of PD patients may also be implied in the potential misjudgment of complex evocative scenes, as fMRI studies on healthy volunteers showed common pattern of activation in the same brain structure following exposure to emotional face expression and IAPS pictures [16].

Failure to discriminate emotional facial expressions may profoundly impact social interactions, potentially complicating relationships and lowering the emotional well-being of PD patients [17,18]. Moreover, the ability to judge the emotional valence of scenes may impact not only social interactions but also the broader context of emotional regulation. PD patients may misinterpret emotional cues from their environment, leading to inappropriate responses to potentially stressful or threatening situations. This misjudgment can increase anxiety and stress levels, affecting overall emotional stability and leading to inadequate coping mechanisms and reduced emotional resilience.

Overall, misunderstandings and miscommunications arising from these deficits can pose significant challenges in the daily life of PD patients, emphasizing the importance of addressing these issues both in research and clinical settings.

Based on these assumptions, this study aimed to obtain a comprehensive view of emotion recognition performances in PD patients by jointly investigating their responses to the Ekman Faces Test and the IAPS stimuli. We expected to confirm the documented difficulty in discriminating emotional facial expressions, while addressing, for the first time, the ability of PD patients to identify the emotional valence of complex emotional scenes. Finally, we evaluated if the ability to recognize emotional facial expressions is associated with the accuracy in classifying the valence of affective scenes.

## 2 Materials and methods

### 2.1 Participants

Forty patients with PD (mean age ± standard deviation, 64.50 ± 8.19 years; range, 44–77 years; 27 men) referring to the Parkinson's and Movement Disorder Center of the San Salvatore Hospital, L'Aquila, Italy, were included in the study. Data collection was performed from March 2021 to March 2022. Inclusion criteria were a diagnosis of idiopathic PD and preserved vision. Exclusion criteria were the presence of a previous history of psychiatric disorders or major concurrent neurological disorders, any previous treatment with DBS and the presence of cognitive impairment. All patients were treated with dopaminergic medications at different dosages. All PD patients were evaluated through the Hoehn and Yahr Staging Scale (H&Y) for disease severity [19] and the Unified Parkinson's Disease Rating Scale (UPDRS), parts 3 and 4. As a control group, a total of 40 healthy controls (mean age ± standard deviation, 64.95 ± 8.25 years; range, 46–78 years; 27 men) matched for age, sex, and education, without a history of psychiatric disorders or of major concurrent neurological disorders, were included.

The Mini-Mental State Examination (MMSE) was used to assess the global cognitive status of participants with a cut-off of ≥26/30 to exclude cognitive impairment. The presence of depressive and anxiety symptoms was assessed using the Beck Depression Inventory (BDI) and the trait–anxiety subscale of the State-Trait Anxiety Inventory (STAI-X2), respectively.

### 2.2 Recognition of facial expressions

The Ekman 60-Faces task [20] represented the standardized tool we used to test recognition of six basic emotions, i.e., happiness, sadness, anger, fear, disgust, and surprise, through photographs of actors displaying facial expressions. Ten photographs were selected for each emotion (10 faces x 6 emotions) and presented, one at a time, on a computer screen. Participants were required to name the expressed emotion for each stimulus by selecting one of six emotional labels. Then, they had to rate the intensity of the emotion expressed on a 1–9 Likert scale (1 = none, 5 = moderate, 9 = extreme). The Ekman 60-Faces test is grounded in extensive empirical research, providing robust validity and reliability in measuring emotion recognition across diverse populations. The test focuses on universally recognized basic emotions, which aligns with our objective of assessing fundamental emotional processing. Moreover, it represents the most commonly used standard set of facial emotion in PD research [9], allowing large comparability of results.

### 2.3 Judgment of affective scenes

Emotional responses to visual scenes were tested using complex pictures from the International Affective Picture System (IAPS) [21]. The IAPS is a widely used database of photographs designed to cover a wide range of affective experiences, from highly positive to highly negative, allowing for a detailed exploration of emotional responses. It provides a standardized set of stimuli that have been extensively validated, ensuring consistency and comparability across studies. Moreover, the availability of normative data on valence ratings for each image, which represented the focus of the present study, facilitated the selection of stimuli to be administered to our samples in order to maximize the content validity of responses. Indeed, following previous studies [6,22], we only used images consistently classified by at least 70 % of normal subjects, excluding stimuli intended to elicit surprise because no item of this category reached the defined consistency level. The resulting 30-stimulus image set included six items for each of the following five emotions: happiness, represented by scenes involving babies or sporting events; sadness, consisting of cemeteries or funeral scenes; anger, including guns or human violence scenes; fear, represented by snakes or spiders; disgust, involving rubbish or rats images. Subjects were required to evaluate the subjectively evoked emotion for each stimulus by choosing one of the five emotional labels and rate the strength of their emotional response on a 9-level Likert scale (1 = not at all, 5 = moderately, 9 = extremely).

### 2.4 Procedure

Participants were seated 60 cm from a computer screen in a quiet room during both behavioral tasks. They were first required to select one of the emotion labels appearing on a single row at the bottom of the screen; the horizontal arrangement of emotion labels changed across stimuli. Then, for the emotion intensity rating, participants had to choose the number corresponding to the perceived intensity from nine numbers horizontally displayed under the row of the emotion labels. All stimuli remained on the screen until participants gave their responses, which were recorded by pressing the corresponding keys on the computer keyboard. No feedback was given about the appropriateness of responses. Each task was preceded by specific verbal instructions and four practice trials (not considered for data analysis). Stimuli presentation was identical for both patients with PD and controls. For data analysis, only ratings on correctly recognized emotions were considered. Both patients with PD and controls completed the two tasks in separate sessions, each lasting 50 min. The task order was counterbalanced across subjects.

All patients received the best medical treatment for their disease at the time of testing and were tested in the “on” state.

### 2.5 Statistical analysis

Differences in sex composition between PD and control groups were estimated using Chi-square test. Furthermore, we evaluated possible differences in age, education years, global cognitive status (MMSE), depression (BDI), and anxiety (STAI-X2) through independent-sample t-tests or Mann-Whitney U tests (in the case of violation of basic assumptions).

Accuracy in recognizing facial expressions and affective scenes (0: incorrect response, 1: correct response) and the intensity ratings of recognized emotions were analyzed at the trial level without averaging over participant trial-by-trial responses. Differences in response accuracy between PD and healthy control groups in the two behavioral tasks were separately evaluated by performing two mixed models using the “lme4” R package. Models included a random intercept for individuals, “experimental group” as a between-subjects predictor (PD, control), “emotional valence” as a within-subjects predictor (six emotions for recognition of facial expressions and five emotions for judgment of affective scenes), and the interaction between “experimental group” and “emotional valence”.

The intensity ratings of recognized emotions for both the behavioral tasks were compared between the PD and control groups using mixed models, including a random intercept for participants and “experimental group” as a between-subjects predictor (PD, control).

All models were fitted using REML, *p*-values were obtained using the Satterthwaite approximation [23], and Holm post hoc comparisons were computed using the “emmeans” R package [24] in the cases of significant interactions.

To exclude possible influences of the different depression and anxiety levels between the two experimental groups (see following “Characteristics of participants” section), control analyses were performed on accuracy measures and emotional intensity ratings, adding BDI and STAI-X2 scores as covariates to the above-described models.

Finally, Pearson's correlation was performed to test the association between accuracy measures in recognizing facial expressions and affective scenes.

All tests were two-tailed, and *p*-values <0.05 were considered significant.

## 3 Results

### 3.1 Characteristics of participants

Demographics (sex, age, education), scores on MMSE, BDI and STAI-X2, and the respective comparisons between PD patients and controls are reported in Table 1. The groups did not differ in sex composition, age, education years, and MMSE scores. Comparisons on BDI and STAI-X2 scores revealed higher depression and anxiety levels in the PD group. Table 1 also shows the mean values of UPDRS-III, UPDRS-IV, and disease duration years for PD participants.

**Table 1:** Characteristics of participants. Results of the comparisons between the experimental groups (Parkinson, Control) on sex composition, age, education level, MMSE, BDI, and STAI-X2 are also shown.

	Parkinson	Control			
	*Mean ± SD*			*Statistic*	*p*
Sex [N (%)]					
Men	27 (67.5 %)		27 (67.5 %)	0.00°	1.00
Women	13 (32.5 %)		13 (32.5 %)		
Age (years)	64.50 ± 8.19		64.95 ± 8.25	−0.24^a^	0.81
Education (years)	11.50 ± 3.21		12.93 ± 3.56	−1.88^a^	0.07
MMSE score	28.35 ± 0.53		28.78 ± 1.35	705.00^b^	0.314
BDI score	5.54 ± 5.75		1.62 ± 2.85	350.50^b^	<0.001
STAI-X2 score	40.24 ± 9.20		31.80 ± 6.59	4.66^a^	<0.001
UPDRS-III score	22.63 ± 10.53				
UPDRS-IV score	3.20 ± 3.75				
Disease duration (years)	5.68 ± 3.17				
H&Y stage	1 (range 1–3)				

*Notes*: ° χ^2^.

*Abbreviations:* SD, standard deviation; MMSE, Mini-Mental State Examination; BDI, Beck Depression Inventory; STAI-X2, trait–anxiety subscale of the State-Trait Anxiety Inventory; UPDRS, Unified Parkinson's Disease Rating Scale, H&Y, Hoehn and Yahr Staging Scale.

a t value.

b Mann-Whitney U.

### 3.2 Recognition of facial expressions

**Fig. 1:**
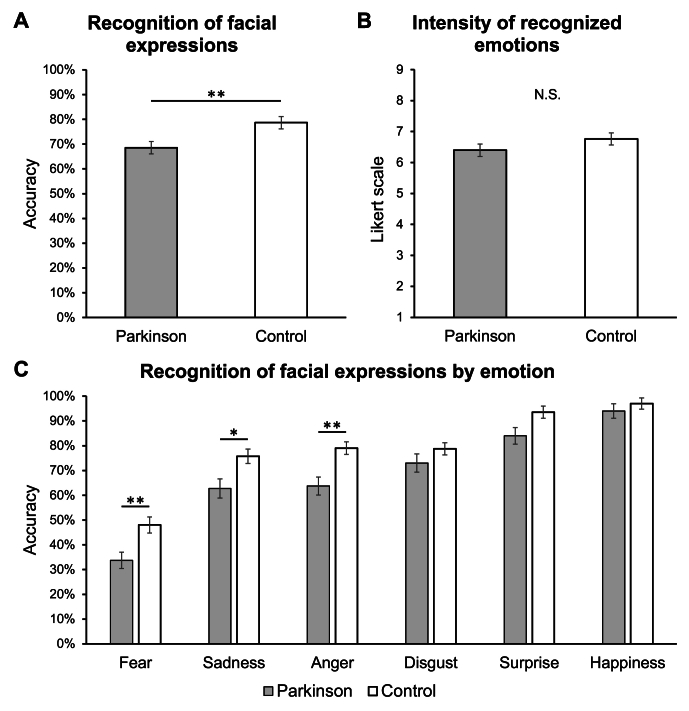
**(A)** Accuracy (%) in recognizing facial expressions in Parkinson and healthy control groups. **(B)** Emotional intensity ratings of recognized facial expression by experimental groups (Parkinson, control). **(C)** Accuracy (%) in recognizing facial expressions of Parkinson and control group depending on expressed emotion. *Notes: The charts reported mean values and standard errors. Significant main effects****(A)****and Holm post hoc comparisons****(C)***are reported with asterisks (*p < 0.05, **p < 0.01).

Analysis on recognition accuracy revealed a significant main effect of the “experimental group” factor (*F*_*1,78*_ = 10.37, *p* = 0.002). As reported in Fig. 1A, PD patients showed lower accuracy (mean ± standard deviation, 68.54 % ± 15.83 %) than healthy control participants (78.67 % ± 12.04 %). Moreover, the main effect of “emotional valence” was significant (*F*_*5,4710*_ = 200.33, *p* < 0.001), indicating differences in accuracy recognition depending on the expressed facial emotion among the overall sample. Holm post hoc comparisons showed significant differences between each of the emotions (all *p* < 0.04), except for the comparison between sadness and anger (*p* = 0.26). The interaction effect between “experimental group” and “emotional valence” factors was also significant (*F*_*5,4710*_ = 3.37, *p* = 0.005), suggesting differences in recognition accuracy between PD and control subjects according to the expressed facial emotion. Holm post hoc comparisons (Fig. 1C) indicated that PD patients showed lower accuracy for faces expressing fear (33.75 % ± 20.84 %, *p* = 0.008), sadness (62.75 % ± 24.60 %, *p* = 0.02), and anger (63.75 % ± 22.95 %, *p* = 0.004) that control group (48.00 % ± 20.53 %, 75.75 % ± 18.66 %, 79.00 % ± 16.14 %; respectively). No difference was detected for faces expressing disgust (PD: 73.00 % ± 23.34 %, Control: 78.75 % ± 15.72 %; *p* = 1.00), surprise (PD: 84.00 % ± 21.10 %, Control: 93.50 % ± 15.45 %; *p* = 0.25), and happiness (PD: 94.00 % ± 18.37 %, Control: 97.00 % ± 14.36 %; *p* = 1.00).

Finally, as reported in Fig. 1B, the analysis did not identify significant differences in the intensity ratings of recognized facial emotion (PD: 6.40 ± 1.35, Control: 6.77 ± 1.12; *F*_*1,78.06*_ = 1.77, *p* = 0.19).

Control analyses adding BDI and STAI-X2 scores as covariates confirmed all the significant main and interaction effects, as well as the significant post hoc comparisons. Moreover, the main analyses were re-applied on accuracy measures after excluding four detected outliers (three in PD and one in the control group), confirming all the above-reported patterns of results.

### 3.3 Judgment of affective scenes

**Fig. 2:**
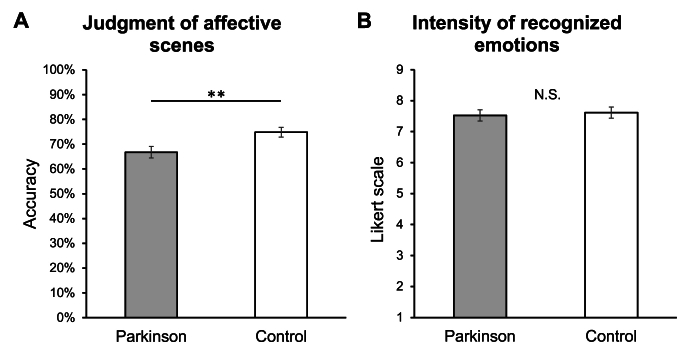
**(A)** Accuracy (%) in recognizing affective scenes in Parkinson and healthy control groups. **(B)** Emotional intensity ratings of recognized affective scenes by experimental groups (Parkinson, control). *Notes: The* charts reported mean values and standard errors. Significant comparisons are reported with asterisks (**p < 0.01).

Analysis on judgment accuracy of affective scenes showed a significant main effect of the “experimental group” factor (*F*_*1,78*_ = 7.01, *p* = 0.01). As reported in Fig. 2A, PD patients demonstrated lower accuracy (mean ± standard deviation, 66.75 % ± 14.59 %) than control participants (74.83 % ± 12.65 %). The main effect of “emotional valence” was also significant (*F*_*4,2312*_ = 57.77, *p* < 0.001), indicating differences in accuracy judgment among the overall sample depending on the emotional valence of the scene. Holm post hoc comparisons indicated that all the emotions differed between them (all *p* ≤ 0.001), except for the comparison between fear and disgust (*p* = 0.59). The interaction effect between “experimental group” and “emotional valence” factors was not significant (*F*_*4,2312*_ = 0.67, *p* = 0.62), suggesting no effects of affective scenes’ emotional valence on the differences in accuracy between PD and control groups.

Finally, as shown in Fig. 2B, the analysis did not detect significant differences in the intensity ratings of recognized affective scenes (PD: 7.53 ± 1.40, Control: 7.62 ± 0.86; *F*_*1,77.01*_ = 0.12, *p* = 0.73). Adding BDI and STAI-X2 scores as covariates did not modify the above-reported pattern of results. The results were also confirmed after excluding three detected outliers (one in PD and two in the control group) from the analysis on accuracy, and two outliers (in the PD group) in the comparison of intensity ratings.

### 3.4 Relationship between facial expression and affective scene recognition

Pearson's correlation analysis on recognition accuracy values between facial expressions and affective scenes indicated significant positive associations in both groups (Parkinson: *r* = 0.57, *p* < 0.001; Control: *r* = 0.57, *p* < 0.001).

**Fig. 3:**
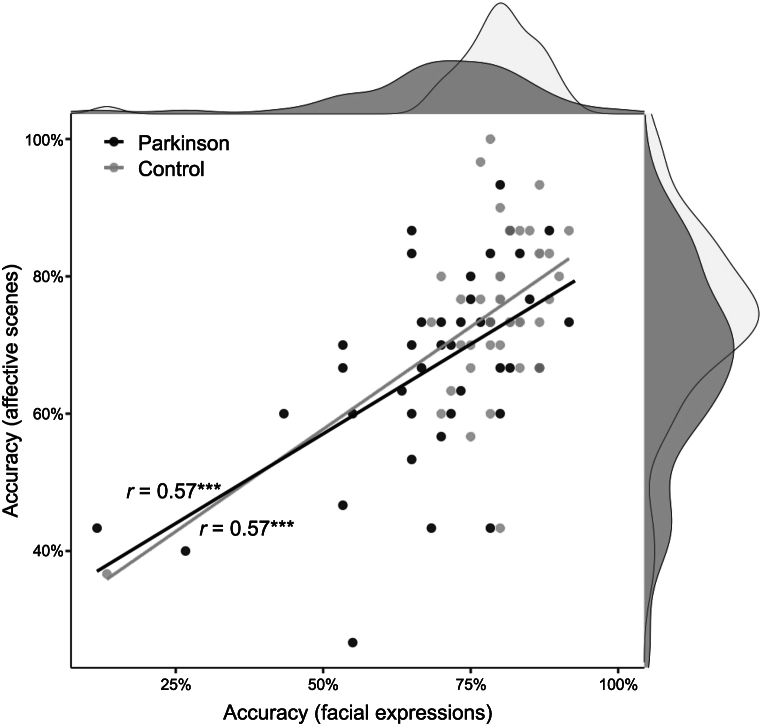
Scatter plot of recognition accuracy values (%) between facial expressions and affective scenes by experimental groups (Parkinson, control). Fitted lines represented the linear relationship between the two accuracy measures in Parkinson and control groups. Pearson's *r* values, significance levels (***p < 0.001), and density plots are also reported.

Higher accuracy in recognizing facial expressions was largely associated with higher accuracy in classifying affective scenes in PD patients and healthy participant group (Fig. 3).

## 4 Discussion

In this study, we presented evidence that patients with PD exhibit reduced accuracy in recognizing emotions from both facial expressions and complex evocative scenes. While previous investigations have focused on the recognition of expressive faces, this study shed light on a little-explored aspect of PD, demonstrating that the disease can also affect the emotional response to scenes selected from the IAPS.

As far as facial expressions are concerned, the findings added to a growing body of evidence that identified dysfunctional emotional processing as a hallmark characteristic of PD [8,9]. Specifically, the analysis showed that the recognition deficit is limited to some negative emotions (fear, sadness, and anger) and did not cover disgusted, surprised, and happy expressions. These results are consistent with current literature, as highlighted by two meta-analyses on this field indicating that the deficit tended to be greater for negative emotions than for the relatively positive ones of happiness and surprise [8,9]. The disproportionate impairment may be related to PD-related dysfunction in specific neural circuits (e.g., ventral striatal dopamine system and amygdala) that are typically involved in the recognition and processing of anger [25], fear [26], and sadness [27].

Functional and structural neural changes in several brain areas and reduced dopamine signals in the mesocorticolimbic pathway have often been invoked to explain impaired facial expression recognition in PD. However, a new hypothesis has emerged in the last few years, proposing that the peculiar deficit in recognizing emotions from human faces may be fueled by the peripheral difficulty of PD individuals in producing congruent facial muscle responses to facial expressions [8]. This idea finds roots in the embodied simulation theory [28], considering the hypomimia that distinctively characterizes PD patients and the role of mimicry in emotion recognition.

On the other hand, analysis of the elicited emotions from IAPS found a generalized impairment in the recognition of emotional responses that extends to complex scenes in the PD group, and this performance is largely correlated with the recognition accuracy for facial expressions. However, the hypothesis based on embodied simulation may not be adequate when it comes to complex scenes that do not involve human faces. The substrate of the generalizability of emotional dysfunction to IAPS scenes may be found in fMRI studies focused on IAPS and expressive faces, which demonstrated similar sets of regions involved in stimulus processing [16,29]. In particular, these studies showed a common pattern of activation in the amygdala in response to emotional faces and IAPS pictures [16,29], supporting the hypothesis of an implication of this brain circuitry in the reported deficit of PD participants. Indeed, current literature consistently showed that the amygdala and its functional connectivity with other brain regions are affected in PD [15], with implications for adequate recognition of affective stimuli [30] and other emotional functions [31].

This study has several strengths. First, it used two different methods to assess emotion recognition. Furthermore, in line with Gray & Tickle-Degnen's meta-analysis [9], supplementary models excluded that the deficit in emotional recognition of faces and scenes is caused by the higher depression and anxiety levels in PD groups [32]. The two groups were matched for age and MMSE, allowing us to exclude confounding effects due to age- or disease-related decline in cognitive functions. Finally, exclusion criteria included a history of DBS, considering that the treatment is associated with impaired facial emotion recognition [33].

Several limitations should also be disclosed. First, PD patients were under levodopa treatment, which may have affected their performance. However, results in this field are contradictory, with studies on patients who had temporarily withdrawn from dopamine-replacement therapy (DRT) that confirmed the deficit in facial emotion recognition [34] and other research that even provided behavioral and [35] neurophysiological evidence [36] for a beneficial effect of DRT on emotion recognition. However, this hypothesis has been challenged by a meta-analytic study [9] that addressed whether medication status moderated the level of emotion recognition difficulties, showing only numerical but not significant differences between participants in a relatively hypodopaminergic state versus those under DRT. Second, our study is exclusively focused on behavioral outcomes. Brain imaging studies are warranted to identify the cerebral structures and pathophysiological mechanisms involved. Third, stimuli used in behavioral tasks consisted of static pictures that poorly reflect the dynamic nature of emotional stimuli in real-life situations. While some studies tested PD patients’ recognition of dynamic facial expressions [37], no study evaluated the emotional response to dynamic scenes in this clinical population. Further research is necessary to fill this gap in the literature by using more ecologically valid stimuli (e.g., movie scenes). Fourth, cognitive functioning evaluation of PD patients has been limited to MMSE administration, and more sensitive instruments for this clinical population should have been adopted [38]. Finally, our behavioral assessment has been focused on emotional valence, not evaluating the elicited arousal following the presentation of Ekman faces and the IAPS stimuli. Both dimensions differentially impact emotional recognition and should have deserved separate evaluations.

In conclusion, the results suggest that individuals with Parkinson's disease have difficulty in recognizing negative emotions from human faces. The emotional processing dysfunction is extended to affective scenes, compromising the adequate emotional response to complex IAPS pictures. These findings are even more relevant when considering that the included patients do not have advanced-stage disease, thus suggesting that emotional processing dysfunctions may develop early following disease initiation. However, the cross-sectional nature of our data collection limits the ability to infer variations over time in emotional recognition capabilities in PD. Future longitudinal studies are warranted to elucidate the temporal dynamics of these non-motor symptoms over the evolution of the PD condition, also addressing how different treatments may interact with the progression of emotion recognition impairments.

The implications of these deficits are significant. Impaired recognition of facial expressions can affect patients’ ability to effectively communicate with others, as facial expressions are an essential aspect of nonverbal communication. This condition can trigger interpersonal distress [17] and problems with psychological functioning [18].

Furthermore, incorrectly interpreting affective scenes may lead to inadequately responding to emotional situations even outside social interaction contexts, impacting PD patients' ability to regulate their emotions. This may be associated with difficulties in coping with stress and other emotional challenges, further impacting their emotional well-being.

It is essential for healthcare professionals and caregivers to recognize this deficit in PD patients and provide appropriate support and interventions, which could involve education and training in recognizing and interpreting emotional stimuli, as well as treatments aimed at limiting the indirect consequences on emotional well-being and quality of life. Some add-on therapeutic interventions for emotional rehabilitation may be suggested in patients with PD. These interventions can include theatre rehabilitation programs based on the representation of emotions: this activity might improve empathy and emotional well-being in PD patients and improve their symptoms in the affective domain. Active music therapy may be also considered a method for effective emotional rehabilitation, simultaneously acting on motor, affective, and behavioral functions. Overall, a multidisciplinary approach involving various healthcare professionals, caregivers and psychologists in the management of patients with PD can provide better outcomes and ensure superior well-being for patients.

## Funding statement

This research did not receive any specific grant from funding agencies in the public, commercial, or not-for-profit sectors.

## Ethics and consent

The study was approved by the Internal Review Board of the University of L'Aquila (n. 05, February 19, 2021) and all the participants gave their written informed consent to participate in the study.

## Data availability statement

The data underlying this article will be shared on reasonable request to the corresponding author.

## CRediT authorship contribution statement

**Federico Salfi:** Writing – review & editing, Writing – original draft, Visualization, Formal analysis, Data curation. **Stefano Toro:** Writing – review & editing, Methodology, Investigation, Data curation. **Gennaro Saporito:** Writing – review & editing, Investigation, Data curation. **Patrizia Sucapane:** Writing – review & editing, Methodology, Investigation. **Massimo Marano:** Writing – review & editing, Methodology, Investigation. **Gianluca Montaruli:** Writing – review & editing, Methodology, Investigation. **Angelo Cacchio:** Writing – review & editing, Methodology, Investigation. **Michele Ferrara:** Writing – review & editing, Methodology. **Francesca Pistoia:** Writing – review & editing, Writing – original draft, Supervision, Methodology, Conceptualization.

## Declaration of competing interest

The authors declare the following financial interests/personal relationships which may be considered as potential competing interests: Co-author is a member of the Advisory Board of the Journal - F.S. If there are other authors, they declare that they have no known competing financial interests or personal relationships that could have appeared to influence the work reported in this paper.
